# Genital Micro-Organisms in Pregnancy

**DOI:** 10.3389/fpubh.2020.00225

**Published:** 2020-06-16

**Authors:** Rashmi Bagga, Parul Arora

**Affiliations:** ^1^Department of Obstetrics & Gynaecology, Post Graduate Institute of Medical Education & Research, Chandigarh, India; ^2^Reproductive Medicine, Nova IVF Fertility, Ahmedabad, India

**Keywords:** genital microbiome, dysbiosis, bacterial vaginosis, candidiasis, Trichomonas vaginalis

## Abstract

The microbiome of the female genital tract may undergo changes in pregnancy due to metabolic, endocrinological, and immunological alterations. These dysbiotic states may cause infections which may ascend upwards to the feto-placental unit or may be seeded hematogenously. These low grade and often low virulent infectious states lead to chronic inflammatory states and maybe associated with adverse maternal and neonatal outcome. Organisms have been isolated from amniotic fluid and placentae from women delivering pre-term; however the possibility of contamination cannot be conclusively ruled out. Common vaginal dysbiotic states often cause symptoms that are overlooked and often untreated. Vulvovaginal Candidiasis (VVC), Bacterial Vaginosis (BV), and Trichomonas Vaginitis (TV) are the commonly occurring dysbiotic states leading to vaginal infective states in pregnancy. With the advent of novel technologies like Next Generation sequencing (NGS), it will soon be possible to comprehensively map the vaginal microbiome and assess the interplay of each microbial state with their effects in pregnancy. This may open new avenues for antibiotic recommendations, probiotics and potential alternate therapies for dysbiotic states leading to pregnancy complications.

## Introduction

It was widely believed that the fetoplacental unit was free of germs and the first exposure of the newborn to microbes occurred during delivery, and not earlier (1). Any bacterial growth in the amniotic fluid or placenta was thought to originate from the lower genital tract and was likely to harm the pregnancy. Bacteria have been shown to traverse the intact materno-fetal membranes and have also been isolated in healthy pregnant women in the amniotic fluid and placentae (2). Recently, microorganisms have been identified in the uterus before conception also (3). It has been suggested that certain obstetrical and neonatal complications are linked to maternal reproductive tract dysbiosis originating from asymptomatic infections, such as chronic endometritis, probably arising prior to conception (4).

Dysbiosis, or change in the microbiome may result from numerous physiological and pathological states. Since ages it has been believed that female genital tract is inhabited predominantly by *Lactobacilli* which prevent infections, and pathologic changes in their profile may make the vagina susceptible to infections which may lead to preterm birth and pregnancy complications. The microbiome may also change with environment, weight, diet pattern and hormonal mileu. Thus, immunological, endocrinological and metabolic changes during pregnancy can cause significant alterations in the microbiome (5).

Most of the earlier knowledge about the vaginal microbiome was derived from studies performing microscopic examination (wet mount and Gram stain) and cultures of swabs collected from the female genital tract. The advent of culture independent techniques like gene amplification and DNA- sequencing techniques have revolutionized metagenomics which can identify the host genome with the inhabiting micro-organisms in the female genital microbiome (6, 7). Traditional Sanger sequencing allowed sampling of the principal species present within a microbiome. Using Next-generation sequencing (NGS) and whole- genome sequencing, it is now feasible to obtain detailed analysis of microbial species present within any site in the body by simultaneous analysis of thousands of sequences (8). This has revolutionized microbiomics and helps in better understanding of eubiosis, which is a healthy balance of the vaginal microbiome, and to determine the various conditions under which the microbiome may be beneficial, are safely tolerated or may be associated with an adverse outcome.

## Alteration of the Microbiome During Pregnancy

Vaginal epithelial mucosa with tight junctions, cervix and thicker cervical mucus plug act as a physiological barrier against invasion of the intrauterine compartment by microorganisms during pregnancy. Throughout the reproductive career, the healthy bacteria and microbiota, predominantly, *Lactobacillus* spp., help in maintenance of a stable vaginal equilibrium and prevent infective states in the healthy reproductive tract. The continued balance of this microbiome during pregnancy aids to the intricate process of sustaining the pregnancy to an adequate gestational age. Despite this, the vaginal microbiome undergoes significant changes during pregnancy by increased stability, a decrease in overall diversity, and predominance of *Lactobacillus species* (8). The vaginal microbiome is quite stable with only shifts of the subtype of *Lactobacillus* and this offers resilience and protection in pregnancy (6). Abundance of *Lactobacilli* in pregnancy results in reduction of vaginal pH and an increased vaginal gland secretions which act as a barrier against pathogenic microbes (9). The major changes in the vaginal microbiome occur during early pregnancy, while during later stages of pregnancy and the puerperium, the vaginal microbiome gets back to baseline, with an increase in diversity, decrease in *Lactobacilli*, and enrichment of bacterial associates (10).

Complex changes in the maternal immune system during pregnancy protect the fetus and mother from infection by promoting development of fetal immunity and preventing fetal rejection by the mother. The genital tract microbiome modulates this immune behavior, however, it is also affected by these immune changes. The interaction between various microbial species and maternal immune system during pregnancy results in an overall increased tolerance to microorganisms. This is mediated by upregulating anti-inflammatory cytokines, initiation of endotoxin tolerance, and suppression of autophagy leading to down modulation of immune response (5). Any alteration in the vaginal microbiota may lead to an increase in pro-inflammatory cytokines with induction of inflammatory cascade and preterm labor. Although infections play a definite role in preterm birth, microbes have also been isolated form gestational tissues of women with normal pregnancy outcomes (11).

The hormonal changes during pregnancy (rising progesterone and estrogen levels) lead to numerous physiological effects which may affect the microbiome composition. In addition, the microbiome can also secrete hormones, highlighting the bidirectional nature of this interplay. However, the direct effects of progesterone and estrogen on the microbiota are not definitely proven (12).

Metabolic changes during pregnancy include changes in energy homeostasis, storage of fat, and hormonal profiles leading to elevated fasting blood sugar levels, insulin resistance, glucose intolerance and weight gain (13). Microbiota is also influenced by changes in metabolism, as noted in obesity, metabolic syndrome, and diabetes. Thus, the metabolic changes occuring in pregnancy are expected to influence the composition of microbiota.

## Composition of the Vaginal Microbiota

The vaginal microbiota is a complex interplay of host cells, symbionts, pathogens with mucosal, endocrinological and immunological factors; and hence it keeps changing throughout a women's life cycle. During childhood, due to low estrogen and thin mucosa, it is mostly dominated by Gram-negative anaerobic bacteria (*Bacteroides, Veillonella, Fusobacterium*), Gram-positive anaerobic bacteri *(Peptococcus, Peptostreptococcus Actinomyces, Bifidobacterium and Propionibacterium)* and certain aerobic bacteria (*Staphylococcus aureus, S. epidermidis Streptococcus viridans, and Enterococcus faecalis)* (14). The vaginal microbiome of prepubertal girls is characterized by less abundance of *Lactobacilli, Gardnerella vaginalis, and Prevotella* (15). Under the estrogenic effect of puberty, the vaginal epithelium thickens and becomes suitable for glucose-fermenting micro-organisms, hence the microbiome in puberty resembles that of adult women being dominated by *Lactobacillus* spp. Other inhabitants include *Escherichia, Staphylococcus, Corynebacterium, Gardnerella, Streptococcus, Mycoplasma, Mobiluncus, Prevotella, and Atopobium* (16). Lactic acid produced by *Lactobacilli* acidifies the vaginal pH (<4.5) thus creating a hostile environment for colonization of pathogenic bacteria and fungi (17). Molecular diagnostic techniques have enabled detection of uncultivated bacteria that were earlier missed by conventional culture techniques. On the basis of the composition of bacterial species in reproductive age women, certain unique microbial community types have also been identified.

As the estrogen levels decline at menopause, the microbiome begins to be composed predominantly by *Lactobacillus crispatus, L. iners, G. vaginalis, and Prevotella* with a less proportion of *Candida, Staphylococcus, Mobiluncus and Bifidobacterium* and hence the vaginal microbiome starts resemblimg pre-pubertal girls (18).

During pregnancy, the presence of normal microbiota helps to protect the genital tract against infection. The vaginal microbiomal patterns of pregnant women delivering at term gestation is different from those of non-pregnant women. Romero et al. (19) used 16sRNA gene sequencing and demonstrated that vaginal microbiome of pregnancy shows more stability than in the non-pregnant state with greater abundance of *L. vaginalis, L. crispatus, L. gasseri, and L. jensenii*. Aagard et al. used NGS to study the changes in vaginal microbiome during pregnancy and found that microbial community varies with gestation and proximity to cervix (8). Freitas et al. also studied the vaginal microbiomes of pregnant women and found less diversity and richness with lesser *Mycoplasma* and *Ureaplasma* load*s, Lactobacilli* abundance and higher bacterial concentration as compared to non-pregnant ones (20). Recent studies have used techniques like RNA gene sequencing to compare the vaginal microbiome of women with Preterm birth (PTB) with those delivering at term and have revealed higher microbial richness and diversity with decreased *Lactobacilli*, abundance of *Gardnerella* and other organisms causing BV (21). Recently, 16srRNA gene taxonomic analysis, cytokine profiling and bacterial genome analysis have been utilized to investigate the dynamics of pregnancy microbiome and specific signature microbiomes have been identified in women with PTB (22).

**Figure 1:**
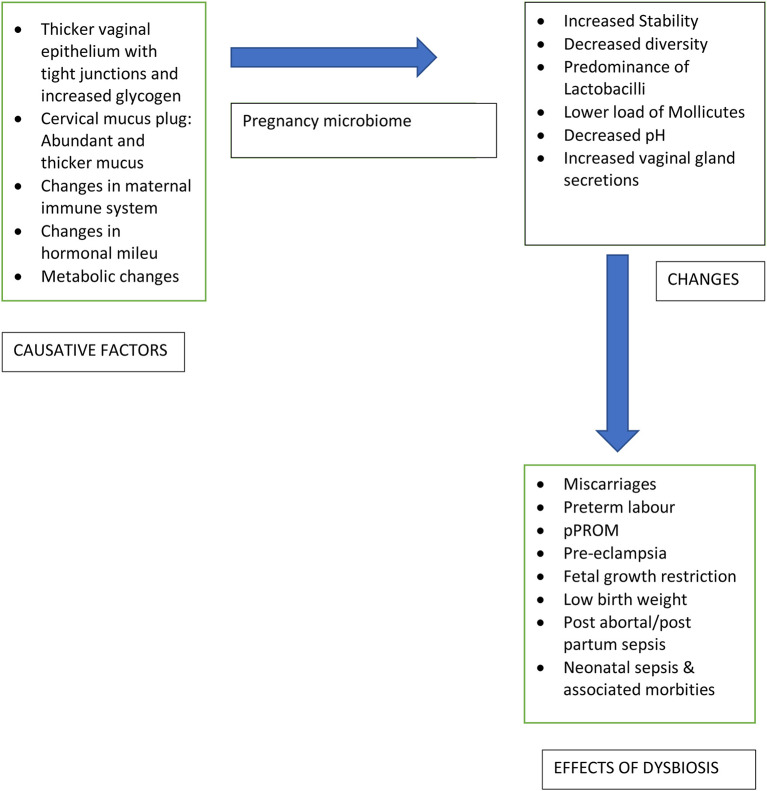
Changes in vaginal microbiome in pregnancy.

An alteration in microbiome composition also called dysbiosis, may make a woman susceptible to genital tract infections which can result in adverse gestational outcomes like preterm onset of labor, preterm prelabour rupture of membranes (pPROM), pre-eclampsia, miscarriage, fetal growth restriction, low birth weight, stillbirth, and neonatal sepsis (23). These infections may ascend from the vagina or cervix or may be seeded haematogenously from non-genital sources like periodontal infections (24–27). Figure 1 highlights the changes in vaginal microbiome with the causative factors and implications of the change.

## Mycobiome and Virome

Researchers have traditionally focussed mainly on the bacteriological aspect of the vaginal microbiome. However, viruses and fungi also contribute to the microbiome and the metagenomics. Hence, authors have termed this as the ‘Mycobiome’ and ‘virome’ (28). Zheng et al. have studied the vaginal mycobiome and found *Candida* and *Saccharomyces* as the predominant species with alterations in the mycobiome with diabetes and pregnancy (29). The vaginal virome has been poorly identified due to difficulties in isolation owing to small viral genomic material and ongoing mutations. However, *Herpesviridae, Papillomaviridae, Polomaviridae* and *Parvoviridae* have been isolated (30).

## Other Genital Microbiota

The vaginal microbiota is the most comprehensively studied microbiome with significant changes in pregnancy and associated with adverse pregnancy outcomes in dysbiotic states. The cervix also has its distinct microbiome. While the ectocervix has stratified epithelial cells resembling the vagina, the pattern of microbiome is also similar and heavily laden; often called cervico-vaginal microbiome. Presence of mucins in the cervical mucus offer antimicrobial activity to prevent ascending infections to the upper genital tract. The endocervix is lined by a single layer of columnar cells with cell junctions and is generally considered sterile, though this has been challenged by identification of microbes using NGS. Distinct microbiota have also been isolated from the endometrium, ovaries and fallopian tubes (30, 31). The association of these microbiota have been studied in women undergoing assisted reproductive techniques. The endometrium has a unique microbiome comprising *Bacteriodetes* esp *Flavobacterium* spp. and *Firmicutes (Lactobacillus* spp) (30, 31). Ovarian follicular fluid and fallopian tubes have shown presence of *Propionibacterium, Streptococcus* and *Lactobacillus*. Alteration in these microbiomes have been associated with recurrent implantation failures (31)

## Dysbiosis and Adverse Pregnancy Outcome

The cause of spontaneous PTB is often unknown, but intrauterine infection is consistently implicated in upto 40% of cases (28). Predisposing factors for infection-related PTB are include intra-amniotic infections, sub-clinical infections and periodontal infections (5, 25, 26). Infections which may cause PTB usually begin in the lower genitourinary tract, ascend upwards and possibly cross the placental barrier. Studies have linked amniotic fluid infection and PTB, postulating that the presence of bacteria within the amniotic cavity is pathological (32). However, other studies have also isolated bacteria from the amniotic cavities of women with term pregnancies (33). Bacteria inhabiting the oral cavity have been also found in amniotic fluid and the placenta without any overt or concomitant inflammation (34). Therefore, their direct causative link is debatable. Infection may occur before conception or early in pregnancy and may also be asymptomatic and undetected (35).

Potential sites of infection include the amniotic cavity with fluid and amniotic membranes, umbilical cord and placenta. Many bacteria have been isolated in such infective states associated with PTB; most common being *Ureaplasma, Mycoplasma, Bacteroides spp., Gardnerella vaginalis, and Fusobacterium* (35). These infections implicated in PTB are associated with organisms with low virulence and early vulnerability in early pregnancy leading to chronic intrauterine infections in the absence of overt clinical signs of infection (5). But, once within the uterine cavity, they induce the release of pro-inflammatory cytokines, prostaglandins, and metalloproteases. These can trigger cervical ripening and shortening, weakening of membranes and rupture, uterine contractility, and pPROM or PTB (5, 28, 30).

## Dysbiosis and Adverse Neonatal Outcomes

Evidence suggest that maternal microbiome acts a bacterial reservoir for microbial seeding of the newborn (36). Studies have evaluated the microbiota of newborn babies delivering vaginally and compared with those born through cesarean section and have found differences. Vaginally delivered infants harbor bacteria resembling the maternal vaginal microbiome, whereas the microbiome of cesarean delivered babies are similar to the microbiota of maternal skin (37). The transmission of vaginal microbiota to the infant may have a protective effect by reduction of Methicillin Resistant *Staphylococcus Aureus* (MRSA) colonization and also by other possible microbes as these site-specific microbial communities develop. Recent evidence also reveal that initial exposure to a specific type of maternal microbiome may influence successive microbial patterns in the gut and other body environments and can influence infant outcomes positively or negatively (38, 39). Microbes in the amniotic-cavity trigger an inflammatory response which makes neonates susceptible to both short-term and long- term consequences, such as early-onset neonatal sepsis (EONS), bronchopulmonary dysplasia, and cerebral palsy (40, 41).

## Amniotic Fluid Microbiota

The microbial pattern in amniotic fluid is characterized by less abundance and low richness and diversity. With the advent of PCR techniques, micro-organisms have also been isolated from sterile amniotic cavities (6). Species commonly isolated are *Proteobacteria, Enterobacteriaceae* (*Enterobacter, Escherichia, Shigella*) (31, 42). Species of *Lactobacillus, Propionibacterium, Staphylococcus, and Streptococcus* have also been detected in amniotic fluids and in the placentae (31). Infant gut microbiological colonization possibly starts *in utero* as microbial species in the meconium is dominated by *Streptococcus, Enterobacteriaceae* (mostly *Enterobacter* and *Escherichia), Lactobacillus, and Propionibacterium* and exhibits similarity with the amniotic fluid and placental microbiome (43). Microbiological colonization of the uterus, amniotic fluid and placenta allows the fetus to exhibit tolerance to bacteria after birth through the phenomenon of priming as it affects the innate gene expression of immune reaction in the fetus and establishment of a healthy microbiome in the newborn (31, 43).

## Placental Microbiota

Both culture and metagenomic techniques have now exhibited the presence of bacteria in the healthy placentae as well (44). Bacteria have also been isolated from placentas of healthy women without chorioamnionitis (45). The presence of placental microbial colonization in a majority of women without apparent adverse perinatal outcomes reinforces that the placental microbiome may be advantageous (33, 46). Whole-genome sequencing has reported that the placenta contains a unique microbiome, somewhat similar to the oral one and not the vaginal microbiome (8, 27). This similarity between the placental microbiota and oral microbiota may be because periodontal infections have also been linked with an increased incidence of pregnancy complications (25–27). It is postulated that bacteria may transmit from the oral cavity to placenta possibly by hematogenous route and not by the ascending migration from lower genital tract. Other studies, however, doubt the presence of a placental microbiome, because bacterial colonization is of low biomass and therefore may be just a contamination (47). Most common isolates from the placental microbiome are *Protobacteria, Bacteroides, Fusobacteria* and *Tenericutes* (31).

Placental colonization if associated with histological evidences of placental inflammation is associated with likelihood to develop pathological effects in neonates like retinopathy or EONS (48, 49).

## Common Genital Infections in Pregnancy

Normal vaginal microbiome comprises of both aerobic and anaerobic bacteria, with Lactobacillus being amongst the predominant microbiota. Lactobacilli provide defense against infections by maintainence of an acidic vaginal pH and ensuring the presence hydrogen peroxide. Decreased concentration of lactobacillus with a concomitant increase in pathogenic microbes may alter the genital tract microbiome and may lead to various infections resulting due to dysbiotic states. Genital infective states often remain inconspicuous during pregnancy, as their signs and symptoms are overlooked as normal symptoms of pregnancy. Additionally, reluctancy to take treatment and reduced tolerability in pregnancy increases the incidence of under-treatment and recurrences. Infections like Vulvovaginal candidiasis (VVC), BV (Bacterial vaginosis), TV (*Trichomonas vaginalis*), HPV (*Human Papilloma Virus*), HIV(*Human Immunodeficiency Virus*), HSV(*Herpes Simplex Virus), Neisseria gonorrhoeae, Chlamydia trachomatis* etc may be consequent to dysbiotic states. HPV is one such infection which infects the basal layer of cervical epithelium and is now believed to be linked to alterations in the vaginal microbiome. Severity of epithelial lesions and progression to cancer has also been associated with certain microbiome patterns due to added oxidative damage and nitrosamines by the microbes (30).

The most commonly encountered dysbiotic conditions during pregnancy namely VVC, BV and TV will be highlighted here.

### Vulvovaginal Candidiasis in Pregnancy

Vulvovaginal candidiasis is a frequent dysbiosis affecting upto 75% of women at least once in their lifetime and 40–45% will have two or more episodes (50). Risk factors include recent sexual activity, antibiotic intake, pregnancy, and immunosuppressive states resulting from conditions such as HIV or diabetes. The risk for developing VVC among healthy women is ~20%, it increases by 30% during the last trimester of pregnancy (51). VVC is caused mainly by *Candida albicans*; however, other species of Candida such as *C.glabrata, C.parapsilosis*, and *C.tropicalis* may be implicated. Candida colonization may disrupt normal microbiome leading to reduction in lactobacilli and an increased pathogenic organisms. Symptoms of candidiasis include non-offensive vaginal discharge, vulval itch, soreness or dysparunea.

In pregnancy, VVC may often be prolonged and associated with more severity in symptoms requiring longer courses of treatment for resolution of symptoms. Although pregnant women are more frequently infected by VVC, they appear to be less symptomatic for vulvovaginitis (52). Topical azoles remain the first line of treatment for vulvovaginal candidiasis during pregnancy (53). Topical Imidazole and vaginal ovules may be used for 2 weeks with repeat courses, if indicated. Oral Fluconazole is avoided in pregnancy as it is reported to be associated with risk of Tetralogy of Fallot, miscarriages and still birth (54–56). Topical imidazoles are safe and effective for the VVC in pregnancy and in breastfeeding as well. Fluconazole levels in breast milk are very low and unlikely to cause harm. Breastfeeding can be continued after a single dose of 150 mg Fluconazole but may be avoided after high doses or repeated courses of fluconazole (57). There is lack of data to evaluate the efficacy of long-term maintenance doses of oral azoles for treating recurrent VVC in pregnancy. Suppressive therapy in pregnancy is generally not offered; thus treating individual episodes with a topical imidazole kept vaginally for 1 week.

The incidence of ascending candidial infection is about 0.8% (58, 59). *C. albicans* needs hyphae formation for local invasion and for crossing intact fetal membranes. It may enter amniotic cavity from the intervillous space or through upward ascent. Chorioamnionitis is rarely linked with VVC in pregnancy or in the presence of foreign bodies such as intrauterine devices or encerclage, and prolonged membrane rupture (59, 60). The mechanism by which VVC during pregnancy may lead to preterm birth remains speculative. Possible release of candidal aspartyl proteinases combined with other virulence factors may promote the degradation of the cervical plug; hence facilitating bacterial ascent in the gravid uterus. There is inconclusive evidence to link the association between VVC and premature delivery or low birth weight (58, 60). Similarly data to recommend treatment of asymptomatic candidial infection in pregnancy to reduce preterm birth is also inconclusive (60).

There is inconclusive evidence to advocate the use of probiotics as an adjuvant therapy in candidiasis in women. It can increase the clinical and mycological cure for a short period and reduce the 1 month- relapse rate at but is ineffective for long-term clinical or mycological cure (61). The same effect cannot be extrapolated to pregnant women and its use is not justified.

### Bacterial Vaginosis in Pregnancy

It is the most common cause of lower genital tract infection in both pregnant and non-pregnant women (62). Bacterial vaginosis is a dysbiotic state in which the vaginal microbiome that is dominated by Lactobacilli is replaced by an overgrowth of certain anaerobic and facultative bacteria. Majority of the women with such an altered microbiome do not elicit any signs or symptoms apart from elevated vaginal pH and microscopic presence of Clue cells studded with bacteria. It may be diagnosed using the clinical Amsel's criteria or Gram staining. Use of PCR and culture techniques for diagnosis is under research settings only. Symptomatic women have a thin milky white foul smelling vaginal discharge. There is no single microorganism implicated in the diagnosis of BV, but rather the alteration of microbiome and presence of different bacteria which characterize this. The bacterial composition of BV include *G. vaginalis, Mycoplasma, Atopobium vaginalis*, and species of *Clostridiales, Mobilincus, Prevotella*, and *Leptotrichia*, but may vary with ethnic groups (58). Bacterial vaginosis may lead to increased susceptibility to STIs and complications after gynecological surgeries. BV during pregnancy has been associated with different obstetric complications such as preterm labor and PTB, pPROM, spontaneous miscarriages, chorioamnionitis, pueperial sepsis, Cesarean wound infections, and gynaecologic complications like postoperative infections and subclinical pelvic inflammatory disease (63–66).

How BV increases susceptibility to adverse pregnancy outcome remains to be confirmed. One hypothesis is that bacteria in the altered microbiome release proteases especially Matrix metalloproteinase-8, sialidases, and other enzymes that disrupt the integrity of the cervical plug (67, 68) their high bacterial concentration in the vagina in BV, leads to possible migration of bacteria into the upper reproductive tract. There, induction of inflammatory cytokines interferes promotes the induction of preterm labor.

Vaginal discharge is commonly reported in pregnancy and may be physiological as well. Women with persisting discharge may be screened for the presence of genital infections. If bacterial vaginosis is detected in a symptomatic pregnant woman, treatment is indicated.

Women with asymptomatic BV are not at risk for pregnancy complications; but those with associated infections or risks of prematurity risks possibly are. Occurrence of BV and its implicated flora are not deleterious *per se*: the interaction with the host, causing an inflammatory reaction, may be implicated for its effects. CDC recommends both oral and topical regimes for treating symptomatic bacterial vaginosis in pregnancy: Metronidazole 500 mg twice daily for 7 days or metronidazole gel (0.75%, 5 g applicator) vaginally for 5 days or clindamycin cream (2%,5 g applicator) intravaginally for 1 week. Alternate regimes include oral clindamycin 300 mg twice daily for 1 week or clindamycin ovules 100 mg vaginally for 3 nights (69). Both metronidazole and clindamycin eradicate upto 85% of BV, but recurrences up to 40–80% are common (70). High recurrence rate may be reduced by follow-up examinations during pregnancy. Likelihood of relapses are not affected by treatment of sex partners (69). Earlier studies had raised concern about metronidazole use in pregnancy but recent evidences by meta analysis have not found any association with metronidazole and teratogenicity or mutagenic effects in newborns. Although metronidazole crosses the placenta, the plasma levels in the neonate are less (71). Few clinicians advise deferring breastfeeding for 12 h only after maternal therapy with (2 g) single dose metronidazole, not with the lower doses. Vaginal clindamycin has also been reported to be safe in pregnancy. Tinidazole should be avoided (69).

Although adverse pregnancy outcomes like PTB, chorioamnionitis, and puerperal endometritis have been linked with BV in few studies, there has been conflicting evidence till date. A recent meta-analysis reported that antibiotic therapy regime could not prevent preterm birth in women with BV (symptomatic or asymptomatic) (72).

Treatment of asymptomatic BV infection in pregnancy in women with risk of preterm delivery (e.g., previous preterm birth) has yielded inconclusive results in studies. (69). A recent review showed a lower preterm birth rate if BV was treated with clindamycin, especially before 22 weeks in women at risk of infection-related preterm birth (73). Similarly, treatment of asymptomatic BV in pregnant women with low risk of PTB is controversial. One study revealed 40% reduction in PTB amongst women using clindamycin between gestation weeks 13–22 (74, 75). Others studies have shown no benefit (76). Therefore, routine screening and treatment of BV in asymptomatic pregnant women at high or low risk for PTB is not recommended (69).

Recent evidences have explored the use of probiotics for the treatment of isolated episodes and recurrent BV. Unfortunately, there is lack of evidence to promote the usage of these agents. One study from China has reported lower recurrence rates in women with recurrent BV using daily vaginal probiotic use compared to placebo (77). Another recent analysis found no harm in administering probiotics (78). A recent trial has shown that that intermittent use of intermittent use of lactobacilli-containing vaginal probiotics may reduce recurrence of BV and may be preferred by both clinicians as they do not affect other body microbiomes or lead to antibiotic resistance (79). Recently, few authors have also reported successful usage of vaginal microbiome transplantation from healthy donors as an alternative for patients with symptomatic, recurrent and intractable BV (80, 81). However, lack of prudent data makes it difficult to extrapolate such treatment modalities in pregnant women.

### Trichomonas Vaginitis (TV) in Pregnancy

Trichomonas Vaginitis is one of the commonest non-viral sexually transmitted infections world-wide. In the absence of true surveillance programs, the epidemiology of TV is not completely studied. It, however, shows variations with ethnic populations and geography. Majority of the women (85%) infected with TV are asymptomatic (82). Symptomatic women complain of diffuse vaginal discharge which is commonly malodorous, yellow-green, associated with dysuria, itching, vulvar irritation and pelvic pain. TV in pregnancy may been associated with PTB and low birth weight (83).

If a woman is symptomatic and tested to have TV infection, treatment is indicated. TV Screening in women with a history of PTB or pPROM is controversial and so is treatment for asymptomatic infection in such cases. Routine screening and treatment of asymptomatic pregnant women with no prior history is not recommended. Some studies have shown benefit with parasitological cure, while few have shown higher rates of PTB in the treated group (84–87).

Metronidazole is considered safe in pregnancy and various meta-analyses have not shown any higher risk of teratogenicity (88, 89). The recommended dose of metronidazole in pregnancy is similar to that for non-pregnant women (2 g) (86, 90). Tinidazole has not been comprehensively studied in pregnancy and remains a class C drug. Lactation should be withheld in women who are administered metronidazole for 12–24 h after the last dose and for 3 days after the last intake of tinidazole to reduce the exposure to the infant (90).

Treatment of *T. vaginalis* infection can provide symptomatic relief in pregnant women and mitigate the sexual transmission to partners, although recurrences are high (91). Although perinatal transmission of trichomoniasis is rare, maternal antibiotics also prevents respiratory and genital infection of the infant (92).

### Alternate Therapy

There is promising evidence from recent studies promoting the use of vaginal microbicidal creams and poly-herbal pessaries in non- pregnant women with vaginal infections (93, 94). Vaginal microbicides help to minimize the mucosal trauma and have minimal adverse effects. Effectiveness of such agents needs to be evaluated in pregnant women. Recent reports have also highlighted the use of vaginal microbiomal transplantation in refractory and recurrent cases of vaginal infections (80, 81).

#### Summary

Microbial colonization of reproductive tract during pregnancy is common and may not always result in complications. Alteration in genital microbiota during pregnancy may involve interactions between infectious agent and the maternal immunity and may lead to inflammatory response and consequent adverse pregnancy outcomes. Women at risk of adverse perinatal outcomes should be timely screened and appropriately treated. Specific interactions between various microorganisms and host mechanisms that promote symbiosis or pathology needs to further evaluated. With the advent of metagenomics and NGS, it may soon be plausible in the future. This may lead to early detection and cure of dysbiotic states with targeted therapies to improve pregnancy outcomes. While antimicrobial therapy has been extensively studied for the cure of vaginal dysbiotic states, novel therapy like probiotics, microbicides and microbiome transplantation appear promising.

## Author Contributions

RB: framework, draft, and correction. PA: draft, correction, and review. All authors contributed to the article and approved the submitted version.

## Conflict of Interest

PA is employed by the company Nova IVF Fertility. The remaining author declares that the research was conducted in the absence of any commercial or financial relationships that could be construed as a potential conflict of interest.
